# Killian Jamieson Diverticulum: A Rare Cause of Dysphagia

**DOI:** 10.7759/cureus.13654

**Published:** 2021-03-02

**Authors:** Lynna Alnimer, Ali Zakaria, Michael Piper

**Affiliations:** 1 Department of Internal Medicine, Ascension Providence Hospital, Michigan State University/College of Human Medicine, Southfield, USA; 2 Department of Gastroenterology, Ascension Providence Hospital, Michigan State University/College of Human Medicine, Southfield, USA

**Keywords:** esophagus, killian-jamieson diverticulum, esophageal diverticulum, esophageal disorder, diverticulum, pulsion diverticulum

## Abstract

Killian-Jamieson diverticula (KJD) and Zenker’s diverticula (more common) share similar pathophysiology but are considered to be different types of phrenoesophageal diverticula.

A 55-year-old female presented to the clinic with chronic dysphagia, halitosis, and regurgitation. Imaging modalities confirmed a Killian-Jamieson diverticulum, explaining her symptoms. She was offered different treatment options and decided to proceed with a less invasive endoscopic approach.

Physicians should be aware of the variable presentations of KJD and the different available treatments as newer techniques are becoming more popular and preferable by patients.

## Introduction

This article was previously presented as a poster abstract at the 2020 Virtual American College of Gastroenterology (ACG) Meeting on October 23-38, 2020.

Killian-Jamieson diverticulum is a rare type of esophageal diverticulum that develops as a protrusion from the lateral wall of the proximal cervical esophagus. It originates in an area of anatomic weakness known as the Killian-Jamieson space, which is inferior to the cricopharyngeus muscle and lateral to the longitudinal muscle of the cervical esophagus [1]. Killian-Jamieson diverticula (KJD) is less commonly encountered in the clinical practice compared to the well-recognized Zenker’s diverticula (ZD), and the differentiation between the two is essential for appropriate treatment. We report a rare case of Killian-Jamieson diverticulum presenting with chronic dysphagia and referred for treatment using an endoscopic approach.

## Case presentation

**Figure 1:**
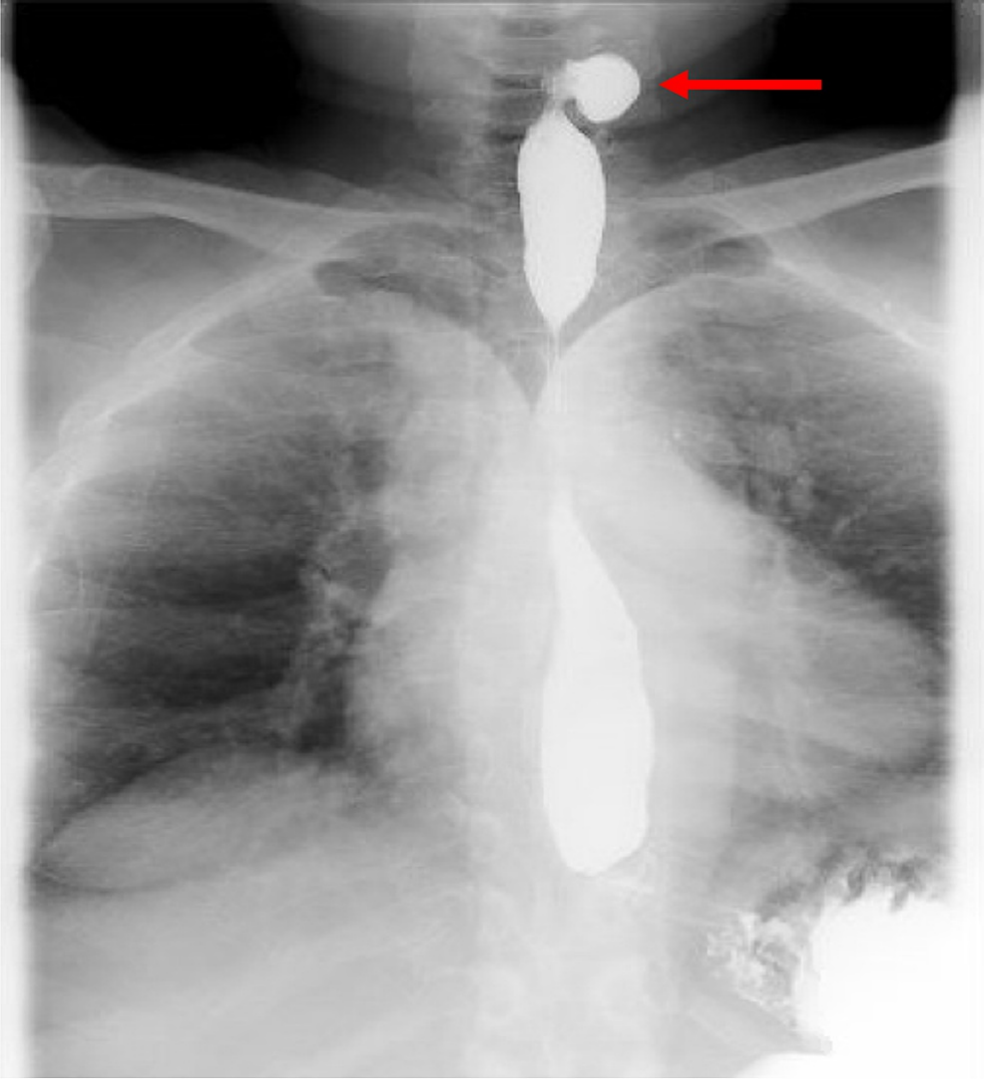
Barium esophagram showing a contrast-filled lateral esophageal out-pouching (red arrow) consistent with Killian Jamieson Diverticulum.

A 55-year-old female with a medical history of Raynaud phenomenon, hypertension, asthma, and seizure disorder was referred to our outpatient gastroenterology clinic complaining of progressive worsening dysphagia to both solids and liquids, halitosis, regurgitation, and neck pain of one-year duration. She had persistent neck pain with radiation to the left ear. Physical examination was unremarkable except for mild erythema of the oropharynx. Subsequent barium esophagram demonstrated a left Killian-Jamieson diverticulum, measuring up to 2.5 cm (Figure 1).

CT scan of the neck revealed a left lateral projecting pulsion diverticulum involving the esophagus at the level of the thyroid cartilage, consistent with a Killian-Jamieson diverticulum. She was offered both surgical and endoscopic (submucosal tunneling diverticulotomy) treatment. However, the patient declined invasive surgical intervention and opted to proceed with endoscopic treatment that was postponed due to the coronavirus disease 2019 (COVID-19) pandemic.

## Discussion

First described by Ekberg and Nylander in 1983, Killian-Jamieson diverticulum remains to be a rare type of pharyngoesophageal diverticulum with a variable clinical presentation [2]. Similar to ZD, KJD is also considered pulsion diverticula or “false diverticula”. In a recent comprehensive literature review of 68 patients by Haddad et al., several epidemiological characteristics were highlighted. The mean age at diagnosis is 58 with female predominance, which fits our patient’s profile. Most cases are unilateral and reported on the left, perhaps suggesting a thinner muscle layer on that side [3]. Nevertheless, bilateral cases have been described in up to 25% of cases [4,5]. Moreover, some cases describe the presence of both ZD and KJD simultaneously, which could suggest possible interrelating pathogenesis [6]. 

Killian-Jamieson diverticulum is usually asymptomatic and is often diagnosed incidentally on radiologic imaging. In symptomatic patients, dysphagia is the most common symptom [3]. Other symptoms include regurgitation, globus sensation, cough, neck pain, neck swelling, hoarseness, and halitosis. On average, larger diverticula are associated with symptoms, with a median size of 2.5 cm [3]. Our patient had a 2.5 cm diverticulum, hence, the multiple symptoms. Interestingly, there is literature reporting a proportion of patients that were diagnosed with Killian-Jamieson diverticulum during thyroid nodule workup, which emphasizes the variety of presenting symptoms [3].

The pathophysiology remains unclear, but weakening in the musculature of the esophagus leading to swallowing dysfunction seems to play a critical role [7]. Several other hypotheses have been described in the literature. One hypothesis is that the Killian-Jamieson diverticulum develops secondary to functional outflow obstruction in the proximal esophagus due to inappropriate contraction of circular muscle fibers [8]. Another theory suggests that symptomatic KJD occurs as a result of esophageal dysmotility secondary to food debris [3]. 

Given the variety of symptoms and presentation, multiple medical and surgical specialties encounter KJD including radiology, otolaryngology, gastroenterology, and endocrinology. This subsequently leads to the use of various diagnostic imaging in the workup of KJD including ultrasound, barium esophagram, esophagoscopy, and CT scan [3]. A barium esophagram is often used to determine the diagnosis of a pharyngeal diverticulum. However, it may be difficult sometimes to distinguish ZD from KJD using barium esophagram solely, especially when the diverticula are large [2-4,7]. Therefore, a CT scan of the neck can be used to delineate the anatomical origin of the diverticulum and the appropriate surgical planning [7]. 

The recurrent laryngeal nerve (RLN) enters the pharynx through the KJ space and is in intimate proximity with the diverticulum. Given the risk of injury, a transverse surgical approach is the preferred option. Unlike in ZD where cricopharyngeus muscle dysfunction is involved in the pathogenesis, and myotomy is usually indicated [9], KJD is located below the cricopharyngeal muscle which may indicate why myotomy is not a fundamental part of management [10]. 

Nowadays, there has been a recent trend towards the use of minimally invasive surgical approaches as they are associated with decreased morbidity, absence of surgical scar, and faster postoperative recovery [10-12]. The endoscopic techniques described vary including the use of monopolar cautery or carbon dioxide laser to separate the intervening septum [8,13-15]. Others used an endoscopic stapler to cut open the diverticula or needle-knife through flexible endoscopy to divide the septum [16,17]. To minimize theoretical RLN injury, the use of traction sutures was also described in repositioning the pouch [18]. Zakaria et al. described a case in an 82-year-old who underwent a successful submucosal tunneling diverticulotomy with no reported complications [19]. Stavropoulos et al. reported good outcomes with submucosal tunneling diverticulotomy in nine patients, compared to direct diverticulotomy [20]. This newer endoscopic method is on the rise given its safety and efficacy, which prompted us to offer her this treatment.

## Conclusions

Killian-Jamieson diverticulum is a rare disease with widely variable presentation and multiple subspecialties encountering this disease. We provided this case report to increase awareness of the clinical findings associated with KJD. Less invasive techniques have been successfully used to treat KJD and can be offered to patients who are at higher risk for surgery or simply opt against an invasive method. Further understanding of the etiology and recommendations on management requires a larger number of patients which remains challenging given the rarity of the disorder.
